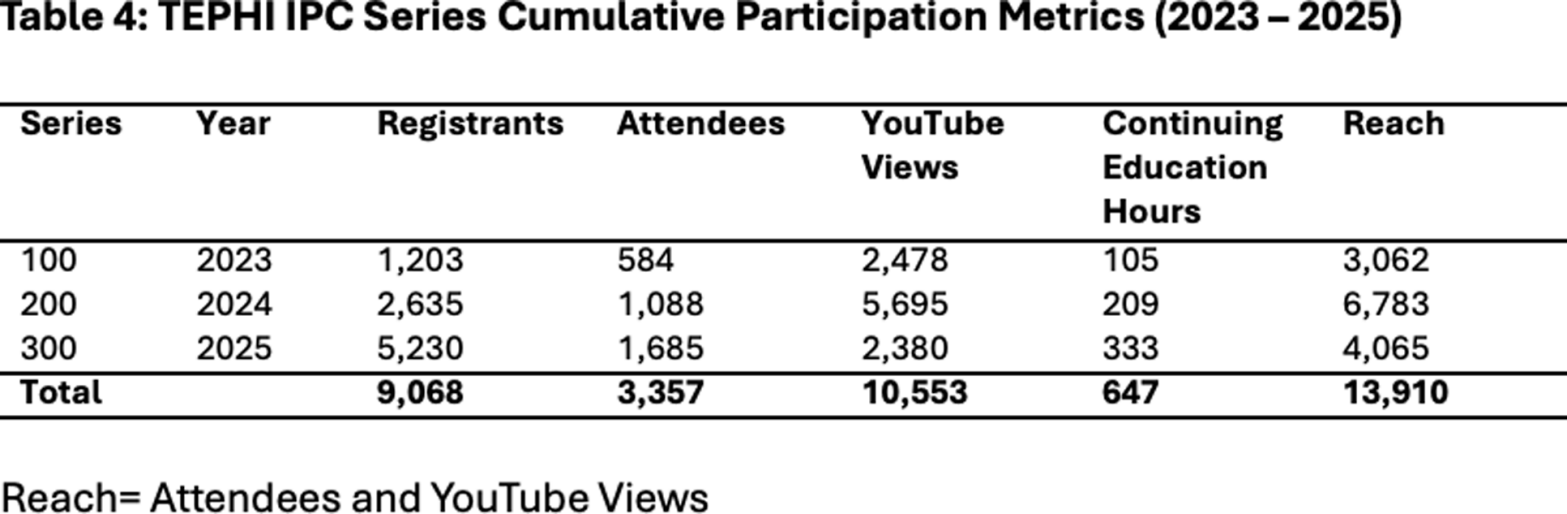# 319 Stewarding the Next Generation: Ten-Year Experience with an Antimicrobial Stewardship Track for Second-Year Infectious Diseases Fellows

**DOI:** 10.1017/ash.2026.10666

**Published:** 2026-06-23

**Authors:** Kayla Ruch, Janelle Rios

**Affiliations:** 1 Becton Dickinson (BD)

## Abstract

**Background:** A resilient healthcare epidemiology workforce is essential to preventing healthcare-associated infections (HAIs), mitigating antimicrobial resistance, supporting antibiotic stewardship, and responding to emerging pathogens. To address statewide gaps in training and preparedness, the Texas Epidemic Public Health Institute (TEPHI) developed a virtual Infection Prevention and Control (IPC) education program. The IPC 300 Series—the third tier of this initiative—focuses on clinical and systems-level applications that strengthen surveillance, outbreak response, environmental risk assessment, and epidemiologic capacity. This evaluation assesses the reach, learning outcomes, and workforce impact of the 300 Series. **Methods:** The 10-module 300 Series (2025) was delivered via Zoom and Microsoft Teams, with recordings available on YouTube for asynchronous access. Registration, attendance, and CE participation were tracked across platforms. Knowledge checks (?80% required) and post-module surveys assessed comprehension and intended practice change using the Kirkpatrick Model (Levels 1–3). Mid- and end-of-series evaluations identified epidemiology training needs, barriers to implementation, and perceived impact on outbreak and stewardship readiness. Descriptive statistics and pairwise t-tests for 2023–2025 trends were conducted in Stata/BE 18.0. **Results:** The 300 Series registered 5,230 individuals, including 1,685 live attendees and 2,070 asynchronous learners—contributing to more than 13,000 learning encounters across three years. Participation represented 49 states and over 20 countries, with 61% from Texas. Continuing education engagement was high (n=325). Knowledge assessment scores (n=511) averaged 92%, exceeding competency thresholds. Modules addressing emerging pathogens, outbreak investigation, environmental risk management, and stewardship-related decision-making generated the highest engagement, reflecting healthcare epidemiology priorities. Post-module surveys demonstrated strong satisfaction (mean 4.7/5.0), with 88% reporting improved understanding and 91% planning to apply epidemiologic principles in surveillance, risk assessment, preparedness, and stewardship-related practices. End-of-series evaluations highlighted persistent gaps in epidemiology and stewardship capacity, including limited staffing, time, and analytic resources. Monthly networking forums (n=323) supported peer learning, case discussion, and practical application of epidemiologic methods. Across 2023–2025, registration doubled and live attendance increased by 55%, demonstrating rising demand for statewide epidemiology-focused IPC training. **Conclusions:** The TEPHI IPC 300 Series strengthens healthcare epidemiology capacity by delivering accessible, competency-based education aligned with surveillance, outbreak response, environmental risk management, and antimicrobial resistance prevention. Light integration of stewardship principles further supports comprehensive infection prevention practice. Strong participation, high learning outcomes, and widespread intent to apply concepts demonstrate the program’s value as a scalable workforce model that advances public health preparedness across healthcare systems.

**Figure:**
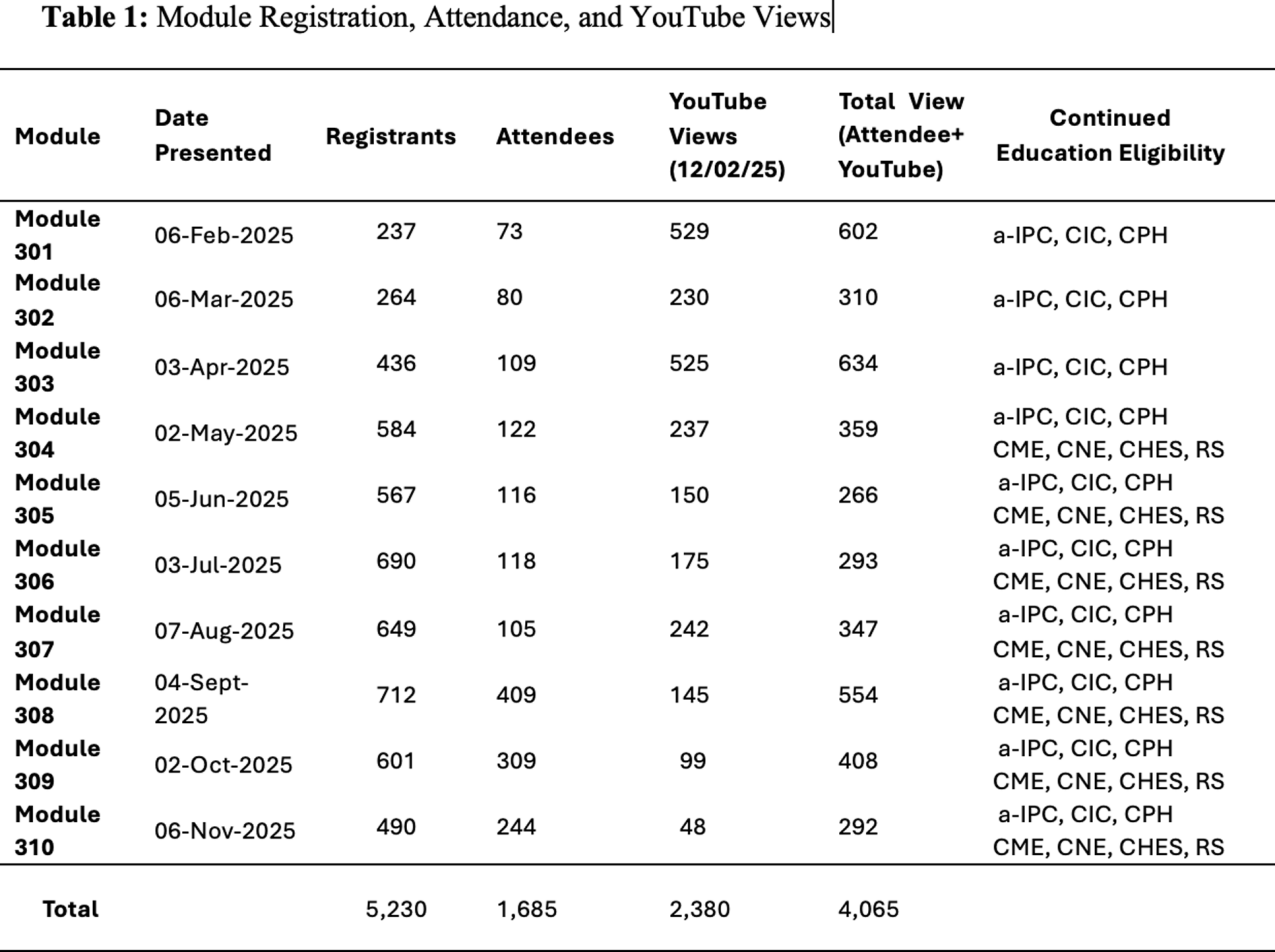

**Figure:**
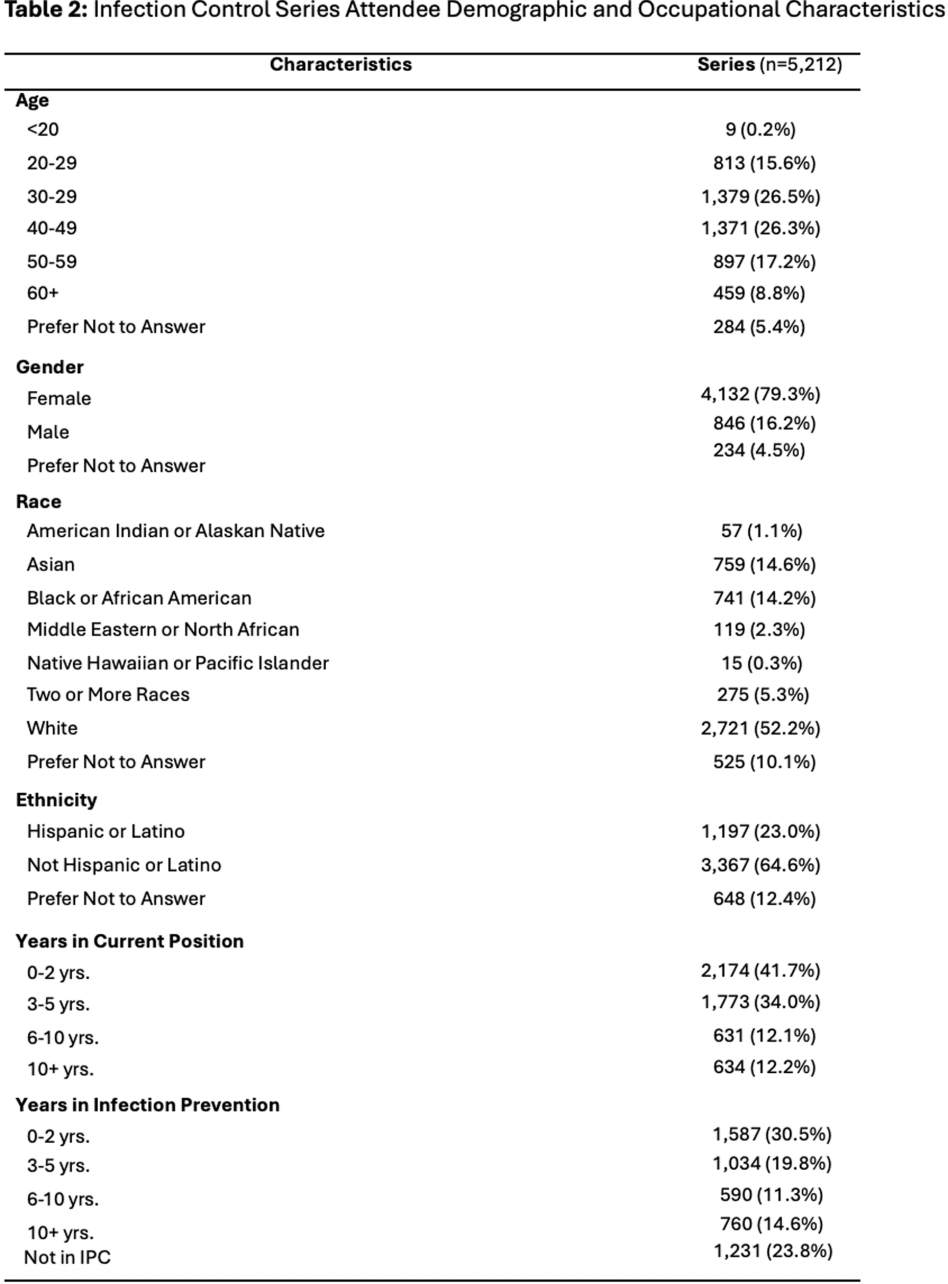

**Figure:**
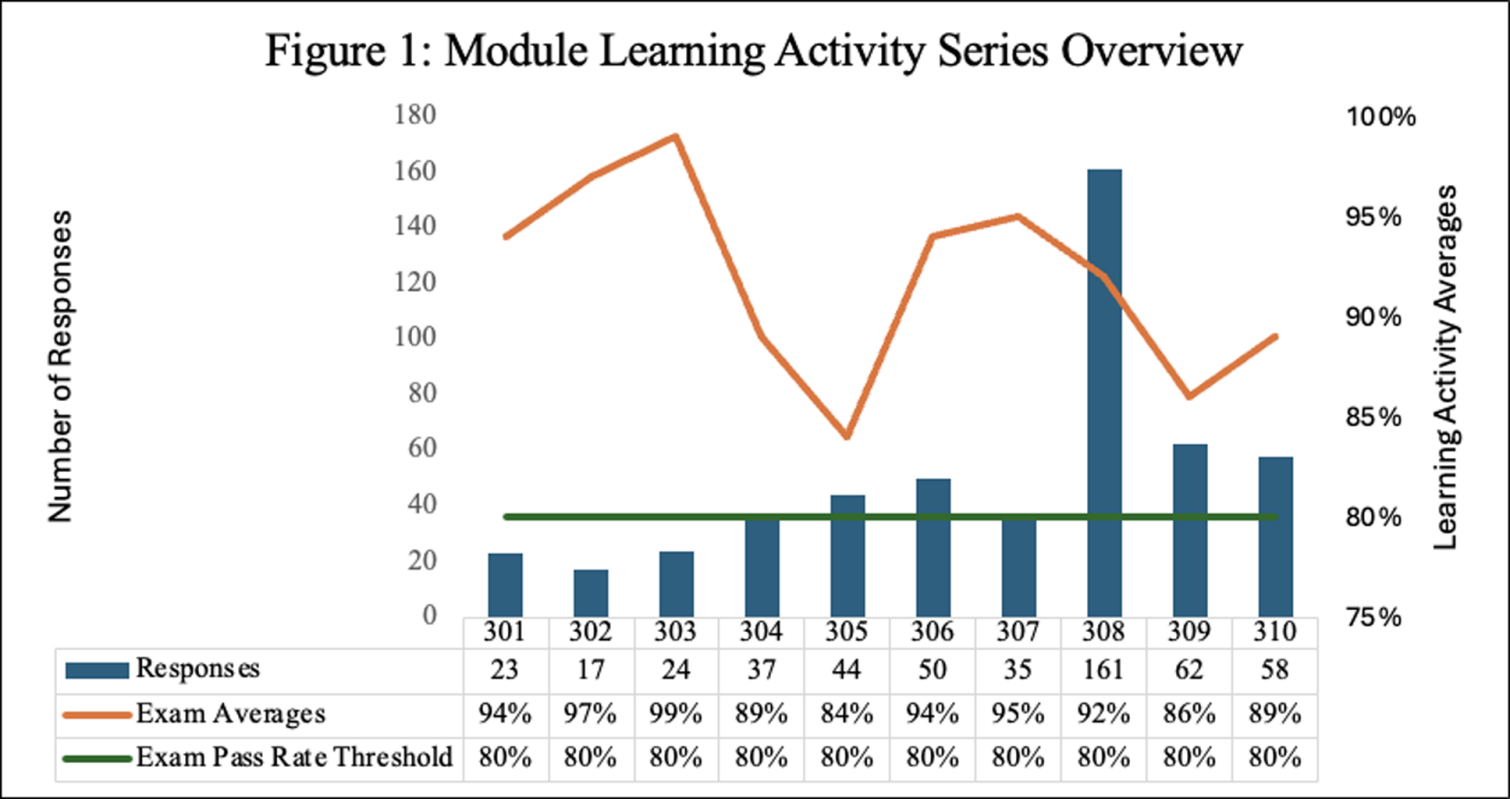

**Figure:**
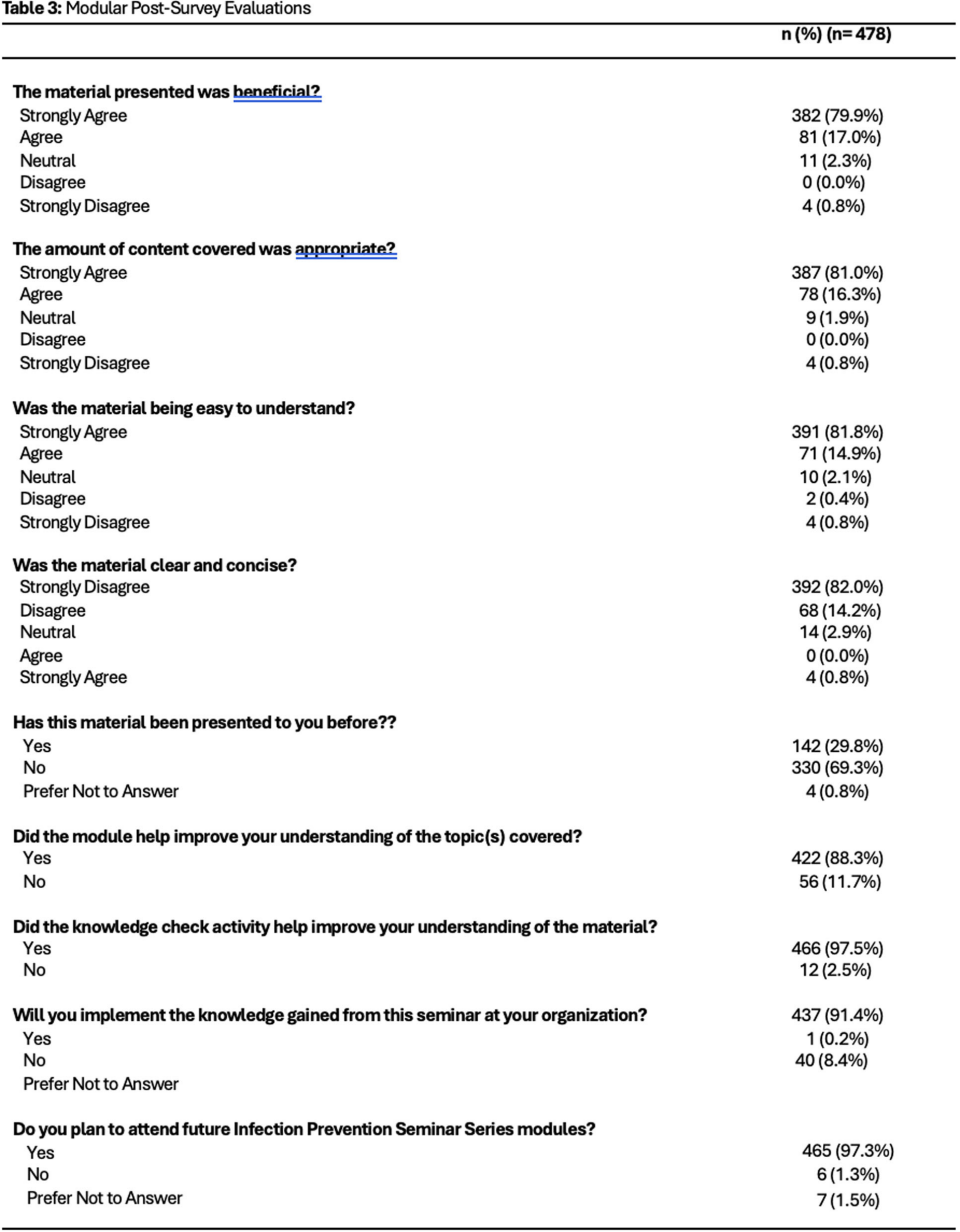

**Figure:**